# Mxc, a *Drosophila* homolog of mental retardation-associated gene *NPAT*, maintains neural stem cell fate

**DOI:** 10.1186/s13578-022-00820-8

**Published:** 2022-05-31

**Authors:** Rong Sang, Cheng Wu, Shanshan Xie, Xiao Xu, Yuhan Lou, Wanzhong Ge, Yongmei Xi, Xiaohang Yang

**Affiliations:** 1 The Women’s Hospital, Institute of Genetics, School of Medicine, Zhejiang University, Hangzhou, 310058 China; 2 College of Life Sciences, Zhejiang University, Hangzhou, 310058 China; 3 Joint Institute of Genetics and Genomic Medicine, Between Zhejiang University and University of Toronto, Zhejiang University, Hangzhou, 310058 China

**Keywords:** Neural stem cell fate, Pros, Mxc, Histone locus body

## Abstract

**Background:**

Mental retardation is a complex neurodevelopmental disorder. *NPAT*, a component of the histone locus body (HLB), has been implicated as a candidate gene for mental retardation, with a mechanism yet to be elucidated.

**Results:**

We identified that *mxc*, the *Drosophila* ortholog of *NPAT*, is required for the development of nervous system. Knockdown of *mxc* resulted in a massive loss of neurons and locomotion dysfunction in adult flies. In the *mxc* mutant or RNAi knockdown larval brains, the neuroblast (NB, also known as neural stem cell) cell fate is prematurely terminated and its proliferation potential is impeded concurrent with the blocking of the differentiation process of ganglion mother cells (GMCs). A reduction of transcription levels of histone genes was shown in *mxc* knockdown larval brains, accompanied by DNA double-strand breaks (DSBs). The subsidence of histone transcription levels leads to prematurely termination of NB cell fate and blockage of the GMC differentiation process. Our data also show that the increase in autophagy induced by *mxc* knockdown in NBs could be a defense mechanism in response to abnormal HLB assembly and premature termination of NB cell fate.

**Conclusions:**

Our study demonstrate that Mxc plays a critical role in maintaining neural stem cell fate and GMC differentiation in the *Drosophila* larval brain. This discovery may shed light on the understanding of the pathogenesis of *NPAT*-related mental retardation in humans.

**Supplementary information:**

The online version contains supplementary material available at 10.1186/s13578-022-00820-8.

## Background

Mental retardation, also known as intellectual disability (ID), is a common neurodevelopmental disorder which has been estimated to be present in 1% of the global population [1]. The etiology of mental retardation is complicated with both external and genetic factors often contributing to this neurological disorder [2]. Chromosomal microarray analysis (CMA) studies, where the copy number variants (CNVs) containing the *NPAT* locus have been categorized as the pathogenic events for mental retardation, implicate *NPAT* as a candidate disease gene [3, 4]. However, no point mutations within *NPAT* gene have yet been reported in mental retardation cases and any potentially related pathogenic mechanisms remain to be elucidated.

*Drosophila* is an ideal model for studying central nervous system (CNS) development and deciphering causes of related diseases. In *Drosophila*, *multi sex combs* (*mxc*) is the ortholog of *NPAT* [5]. NPAT/Mxc is a key component of histone locus body (HLB) [6–8]. Studies have shown that NPAT/Mxc acts as the scaffold protein of the HLB required for HLB assembly and transcription of canonical histone genes [5]. The N-terminal of NPAT/Mxc is crucial for self-interaction, while the C-terminal is required for recruiting other members of the HLB [9, 10]. NPAT/Mxc is phosphorylated by cyclin E/Cdk2, such phosphorylation being a key factor in the formation of phase separation allowing the formation of HLB to be precisely and dynamically regulated [11, 12]. Several studies on *NPAT/mxc* mutations have been published with the mutations of *NPAT*/*mxc* leads to the failure of HLB formation and in the block ageing of histone transcription in cultured cells [10, 11]. The chromosome instability caused by *mxc* mutations affects *Drosophila* male meiosis [13]. In *Drosophila* larval hematopoietic tissue, depletion of *mxc* leads to tumor formation [14, 15]. Based on these reports, *NPAT /mxc* appears to prevent chromosome instability and acts as a tumor suppressor gene. How a “tumor suppressor” *NPAT*, when its function is defective, leads to human mental retardation remains a key question. Our study focuses on the specific role of NPAT/Mxc in the development of *Drosophila* CNS, and reveals its possible mechanisms related to mental retardation.

*Drosophila* CNS is derived from neuroblasts (NBs), also known as neural stem cells [16]. The NB divide asymmetrically producing a self-renewing NB and a smaller ganglion mother cell (GMC). The GMCs further undergo differentiation and divide terminally, giving rise to two neurons [17, 18]. At the pupal stage, all NBs terminate their cell fate and exit the cell cycle [19, 20]. No mitotic NBs remain detectable in the central brain or ventral nerve cord of adult flies [21]. Deadpan (Dpn) and Prospero (Pros) are two important transcription factors that regulate the stemness and differentiation of NBs, and are commonly used as markers to identify NBs and their progeny [22–24]. Dpn is expressed in all NBs and mature INPs, but not in immature INPs or GMCs [25]. Ectopic expression of Dpn in immature INPs leads to dedifferentiation and tumor formation [23, 26]. Pros, as a cell fate determinant, is expressed in all NBs but only localized at the basal cortex during mitosis in the larval central brain. At telophase, Pros is exclusively segregated into the GMCs [27, 28]. Soon after the formation of GMCs, Pros enters the nucleus and acts as a transcription factor that inhibits stemness and promotes differentiation [24, 29]. At the early pupal stage, during which all CNS neurons are developed, Pros starts to accumulate in the nucleus of NBs. These NBs with nuclear Pros divide symmetrically to generate two equal sized daughters and terminate the NB cell fate [30, 31].

In the present study, we identify that Mxc/NPAT functions to maintain the proliferation of NBs and their progeny in *Drosophila* larval brains. The lack of Mxc leads to decreased transcription levels of histones and increased DNA double strand breaks (DSBs), resulting in premature termination of NB cell fate and impaired NB self-renew potential. Knocking down *mxc* also inhibits the differentiation of GMCs. These abnormalities drastically reduce the numbers of neurons in the central brain, eventually leading to neurological defects. Our data also show that in the absence of Mxc, autophagy is elevated and seems to protect against the formation of Dpn^+^ (differentiation-halted) GMCs. These data shed a light on the possible pathogenesis of human mental retardation disease caused by *NPAT* mutations.

## Results

### Mxc deficiency leads to a massive loss of neurons and locomotion dysfunction in adult flies

**Fig. 1.**
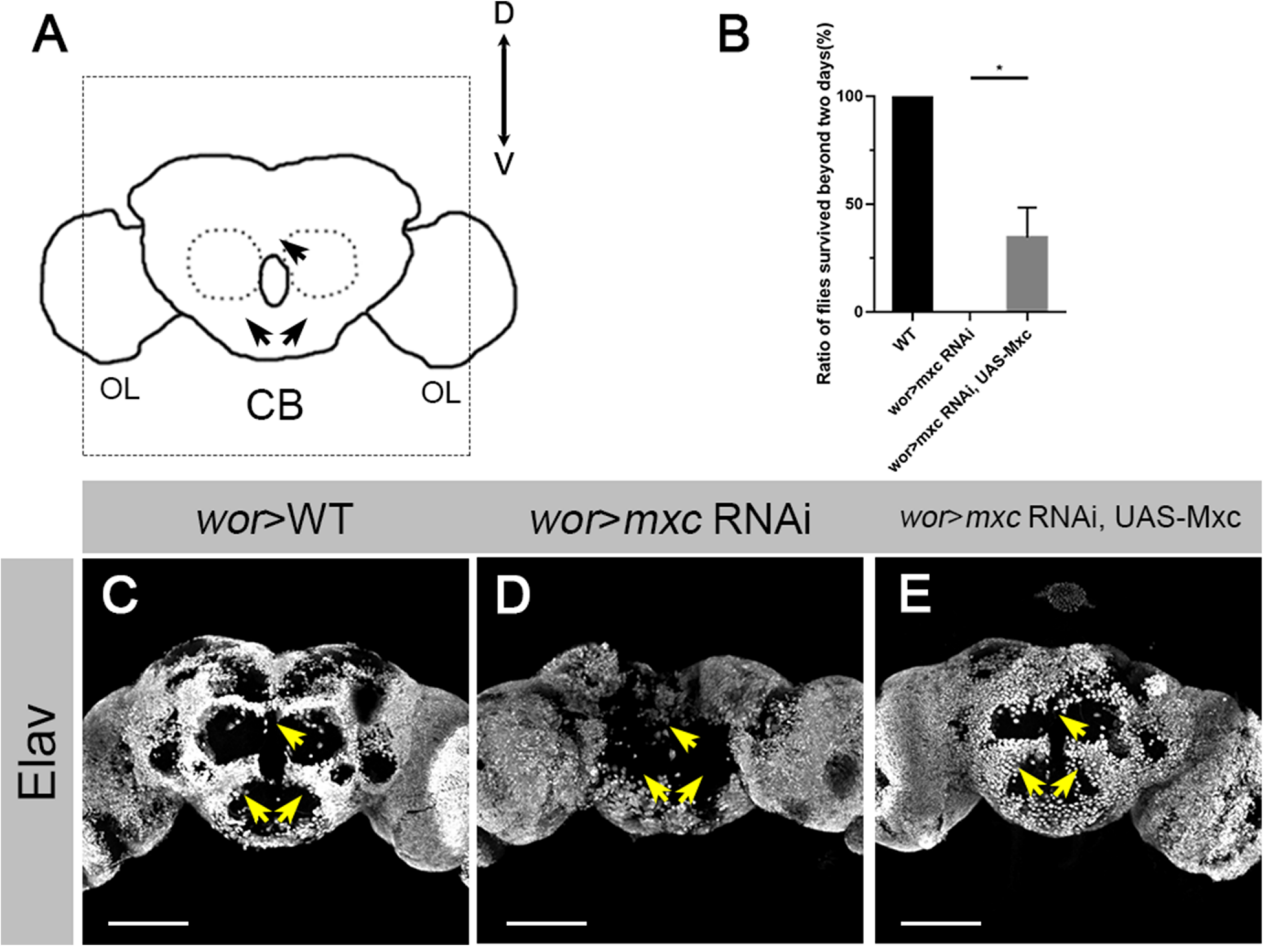
Knocking down *mxc* causes neuronal defects. **A** Schematic of the structure of *Drosophila* adult brain, including central brain (CB) and optic lobes (OLs). **B** Statistics on survival rate of *wt*, *mxc* RNAi and Mxc rescue flies after eclosion. The data are plotted as mean ± SD. *p < 0.05 using a Student’s *t* test, *p* = 0.0124. Numbers of flies counted N = 99, N = 107, N = 106, respectively. **C**–**E** Z-axis projection of adult central brains of *wt* (**C**), *wor* > *mxc* RNAi (**D**), *wor* > *mxc* RNAi, UAS-Mxc (**E**), labeled by anti-Elav. Elav is a maker of differentiated neurons. Elav^+^ cells were distributed throughout *wt* central brains (arrowheads; **C**), but were missing in *mxc* RNAi central brains (arrowheads; **D**). Overexpressing Mxc in *mxc* knockdown background rescued the phenotype (arrowheads; **E**). Scale bars: 100 μm

To explore the potential functions of Mxc in CNS development, we employed a NB specific RNAi knockdown approach. The *mxc* RNAi lines were driven by *wor*-GAL4. *mxc* knockdown flies survived till the early adult stage, but all stayed on the bottom of the vials after eclosion without climbing ability and exhibited akinesia. These flies then died within the first one or two days of adulthood (n = 107; Fig. 1B). Overexpression of Mxc in *mxc* knockdown flies partially rescued this phenotype, in which about one third of the flies survived beyond two days (34.58%, n = 106; Fig. 1B).

We dissected brains from the adult flies within one day of eclosion and stained with anti-Elav, a pan-neuronal marker. Compared to the *wt* (Fig. 1A). The *mxc* knockdown flies showed a massive loss of neurons in the central brain (arrowheads, Fig. 1D). The exogenous overexpression of Mxc in larval NBs largely rescued this phenotype (Fig. 1E). It is interesting to note that no obvious abnormality of brain development was observed when Mxc was overexpressed in larval brain NBs of wt flies. These observations indicate that Mxc plays a vital role in the CNS development. *mxc* knockdown in larval NBs causes the loss of neurons in the adult brains, leading to locomotion dysfunction and early lethality of adult flies.

To further evaluate the important role of Mxc in CNS development, we examined the genetic mutant of *mxc*. *mxc*^*16a − 1*^ is a mutant line of *mxc* (Obtained from BDSC), containing a deletion of 4 bp (TTCG) at residues F1823 of Mxc and resulting in a frameshift at the C-terminal [5]. Hemizygotes of *mxc*^*16a − 1*^ die at late larval or early pupal stage [32]. NB specific overexpression of exogenous Mxc in hemizygotes of *mxc*^*16a − 1*^ prolonged the survival time of the mutant animals to the late pupal stage (80.26%, n = 101) where a small percentage (10.82%, n = 101) of the flies even survived to adulthood without obvious mobility problems. This rescue data agrees well with the *mxc* RNAi result and suggest that Mxc is critical for CNS development.

### **Knockdown of*mxc*results in premature localization of Pros in the nucleus of NBs**

**Fig. 2:**
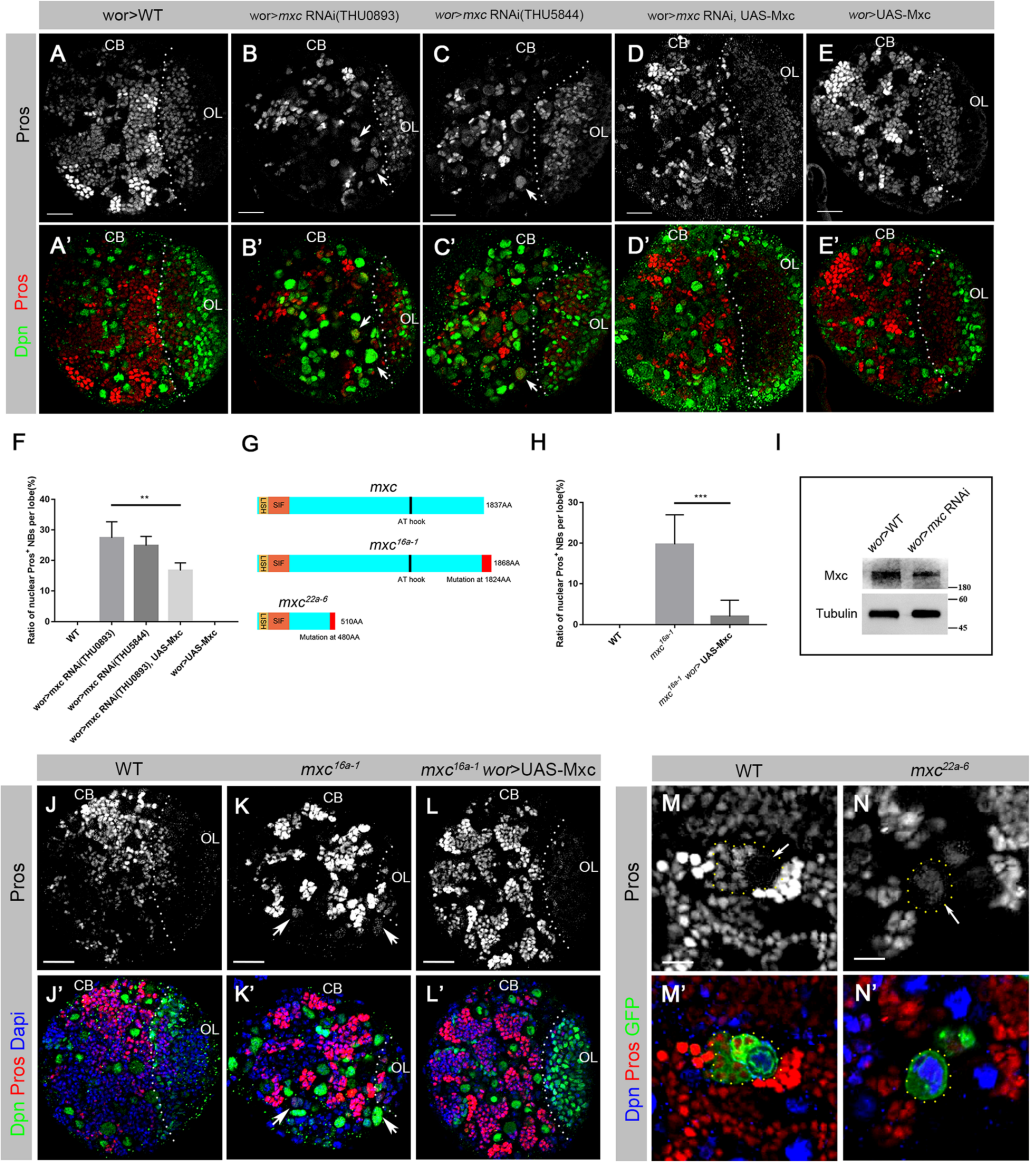
Lack of Mxc leads to premature accumulation of nuclear Pros in NBs.  **A**–**E’** Confocal images of the third-instar larval brains of *wt* (**A**–**A’**), *mxc* RNAi (THU0893, THU5844) (**B–C’**), *mxc* RNAi with UAS-Mxc (**D–D’**) and UAS-Mxc (**E**–**E’**), labeled by anti-Pros (red) and anti-Dpn (green). In *wt* larval central brains, no nuclear Pros was detected in the NBs. Pros (arrowheads) was detected in the nucleus of NBs in *mxc* RNAi lines. This phenotype was rescued by UAS-Mxc. No phenotypes were detected in UAS-Mxc lines. All of these lines were driven by *wor*-Gal4. CB represents the central brain region, and OL represents the optical lobe region. Scale bars: 20 μm. **F** Statistics on nuclear Pros in the NBs in larval central brains of *wt*, *mxc* RNAi and Mxc rescue. The data are plotted as mean ± SD. **p < 0.01 using a Student’s *t* test, *p* = 0.0010. Numbers of NBs counted N = 360, N = 740, N = 69, N = 108, N = 187, respectively. **G** Schematic of Mxc protein structures in *wt*, *mxc*^*16a − 1*^, and *mxc*^*22a − 6*^. The 1837–amino acid Mxc protein contains a LisH domain (amino acids 6–38) and a SIF domain (amino acids 39–185) at the N-terminal, as well as a AT-hook motif at residues 1523–1535. *mxc*^*16a − 1*^ contains a deletion of 4 bp (TTCG) at the F1823 residue of Mxc, resulting in a frameshift at the C-terminal. It encodes an 1868–amino acid protein. *mxc*^*22a − 6*^ encodes a nonsense mutation at the N480 residue of the Mxc protein, resulting in a truncated protein containing 510 amino acids [10, 32]. **H** Statistics on nuclear Pros in the neuroblasts in larval central brain of *wt*, *mxc*^*16a − 1*^ and Mxc rescue. The data are plotted as mean ± SD. ***p < 0.001 using a Student’s *t* test, *p* = 0.0007. Numbers of neuroblasts counted N = 360, N = 373, N = 153, respectively. **I** The protein levels of Mxc were decreased in *mxc* knockdown larval brains compared to *wt*. MPM-2 antibody was used to recognize Mxc signals in this experiment. (**J**–**L’**) Confocal images of the larval brains of *wt* (**J–J’**), *mxc*^*16a − 1*^ (**K–K’**), and *mxc*^*16a − 1*^ with UAS-Mxc (**L–L’**) at 120 h AEL, labeled by anti-Pros (red), anti-Dpn (green), and Dapi (blue). Pros (arrowheads) was detected in the nucleus of *mxc*^*16a − 1*^ larval NBs (**K**). This phenotype was well rescued by worniu driven UAS-Mxc (**L**). Scale bars: 20 μm. (**M–N’**) MARCM clones of *wt* (**M–M’**) and *mxc*^*22a − 6*^ (**N–N’**) in third-instar larval brains, labeled by anti-Pros (red), anti-Dpn (blue) and anti-GFP (green). Nuclear Pros (arrowhead) was not detected in *wt* NBs (**M**), but was detected in *mxc*^*22a − 6*^ NBs (**N**). Numbers of clones observed n = 3, n = 3, respectively. Scale bars: 10 μm

The massive loss of neurons in adult central brains could be due to defects in NBs and GMCs in larval brains. This prompted us to investigate the NB cell fate maintenance in *mxc* knockdown larval brains. In NBs, nuclear localization of Pros is recognized as a signal for the termination of stem cell fate [20, 30, 31].We observed that NB specific *mxc* knockdown with *wor*-GAL4 led to a premature accumulation of Pros in nuclei of the NBs of the third-instar larval central brains (arrowheads; Fig. 2B). To ensure the observed phenotype was not an off-target effect, a second *mxc*-RNAi line (THU5844, obtained from Tsinghua *Drosophila* Center) was used and the rescue experiment was conducted as well with a *mxc* overexpressed line (Fig. 2A–F). The efficiency of RNAi knockdown was confirmed using Western blotting analysis with MPM-2, an antibody recognizing cyclin E/Cdk2–dependent phosphorylation sites within Mxc [5] (Fig. 2I). The brain samples were double-labeled with anti-Dpn, a stem cell marker [23, 24] and anti-Pros. In *wt* third-instar larval brain NBs, Pros was not detected in the nuclei of NBs (Fig. 2A). In contrast, we observed nuclear Pros in NBs in both *mxc* RNAi lines (THU0893: 27.51%, n = 740; THU5844: 24.84%, n = 69; Fig. 2B, C, F). Overexpression of exogenous Mxc in *mxc* knockdown background partially rescued the phenotype (16.67%, n = 108; Fig. 2D, F). The overexpression of Mxc alone in *wt* NBs did not result in any phenotypes (Fig. 2E).

We further confirmed the phenotype using two *mxc* mutant lines, *mxc*^*16a − 1*^, and another *mxc*^*22a − 6*^ (From BDSC; Fig. 2G). *mxc*^*22a − 6*^ contains a nonsense mutation at residues N480 of the Mxc protein, resulting in a truncated protein [5]. The hemizygotes of *mxc*^*22a − 6*^ die as first or second instar larvae [32]. As expected, nuclear Pros was observed in the NBs of *mxc*^*16a − 1*^ third-instar larval brain at 120 hours AEL (After Eggs Laying; 19.71%, n = 373; Fig. 2J–L’, H), and the phenotype was effectively rescued by overexpressing exogenous Mxc (2.00%, n = 153; Fig. 2L, H). Since the hemizygote of *mxc*^*22a − 6*^ died at the first or second instar larval stage, we performed MARCM analysis on *mxc*^*22a − 6*^ (Fig. 2M, N’) [33]. The immunofluorescent staining also showed nuclear Pros in the *mxc*^*22a − 6*^ homozygous lineages (Fig. 2N). This was consistent with the RNAi experimental data. We employed the THU0893 line (referred as the *mxc* RNAi line) for the following experiments.

### Mxc maintains the NB number and cell fate in larval central brains

**Fig. 3:**
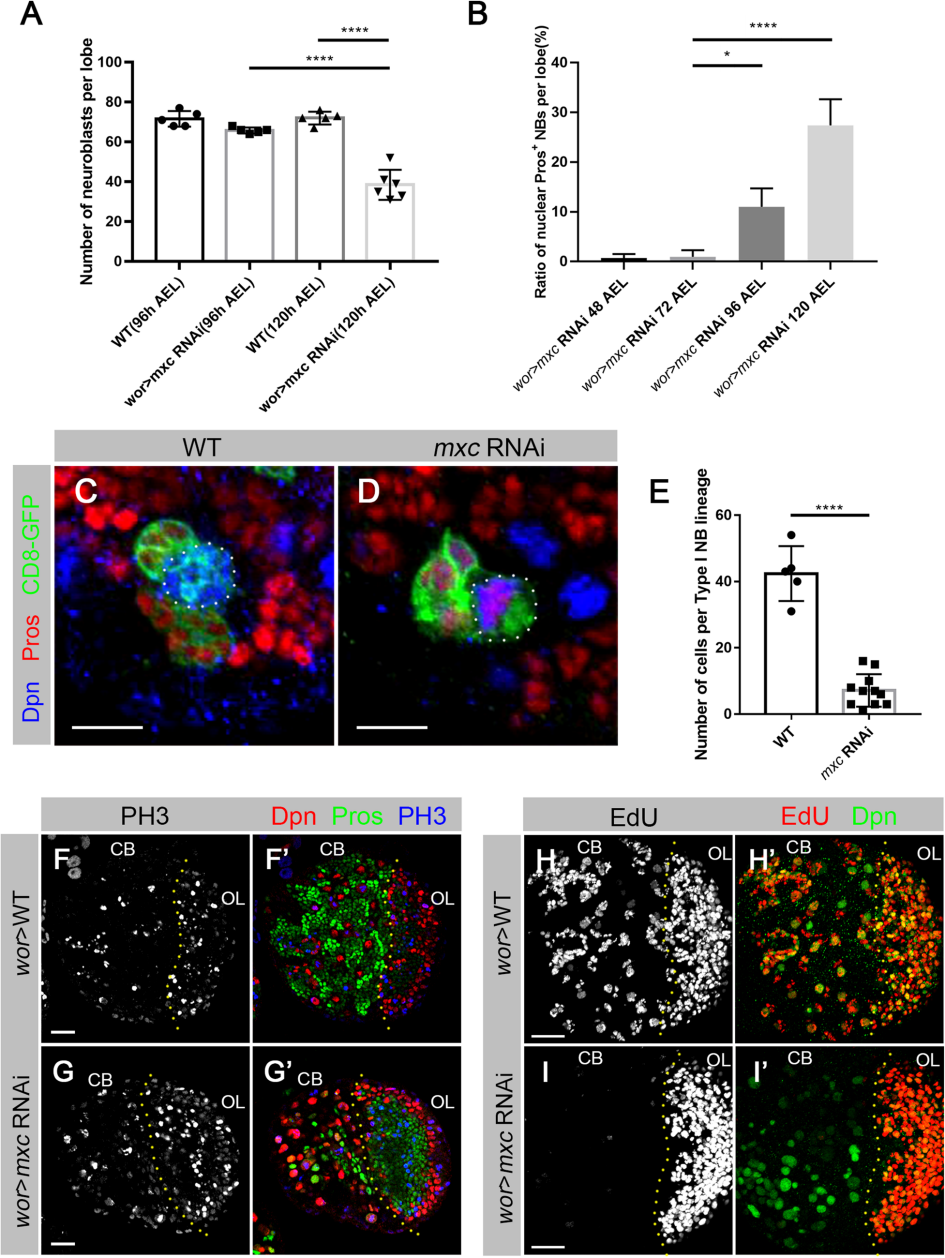
Mxc is required for NB proliferation and cell fate maintenance. **A** Statistical analysis of the numbers of NBs at different developmental stages (96 and 120 hours AEL) in *wt* and *mxc* RNAi brain lobes. Dpn^+^ cells larger than 8µm in diameter were considered NBs [25]. The data are plotted as mean ± SD. ****p < 0.0001 using a Student’s *t* test, *p* = 2.555E–05 and 7.502E–06, respectively. Numbers of brain lobes counted N = 5, N = 5, N = 5, N = 6, respectively. **B** Statistics on nuclear Pros in the NBs in *mxc* RNAi larval central brain at various developmental stages of 48, 72, 96 and 120 hours AEL (After Eggs Laying). The data are plotted as mean ± SD. *p < 0.05 and ****p < 0.0001 using a Student’s *t* test, *p* = 0.0160 and 3.574E–06, respectively. Numbers of NBs counted N = 239, N = 112, N = 310, N = 740, respectively. **C, D** Third-instar larval brains containing Type-I NB FLP-out clones of *wt* (**C**) and *mxc* RNAi (**D**), labeled by anti-GFP (green), anti-Dpn (blue) and anti-Pros (red). Doted circles mark the location of the NBs. *wt* clone showing one large NB with dozens of small daughter cells (**C**). *mxc* RNAi clone showing one large NB and fewer daughter cells in one lineage (**D**). Scale bars: 10 µm. **E** Statistical analysis of the numbers of cells in each *wt* lineage and *mxc* RNAi lineage. The data are plotted as mean ± SD. ****p < 0.0001 using a Student’s *t* test, *p* = 3.620E-08. Numbers of clones counted N = 5, N = 11, respectively. **F**–**G’** Third-instar larval brains of *wt* (**F**–**F’**) and *mxc* RNAi (**G**–**G’**), labeled by anti-Dpn (red), anti-Pros (green) and anti-PH3(blue). No differences of PH3 signals in NBs between *wt* (**F**) and *mxc* knockdown (**G**) were observed. Scale bars: 20 μm. **H**–**I’** Z-axis projection of third-instar larval brains of *wt* (**H**–**H’**) and *mxc* RNAi (**I**–**I’**), labeled by EdU (red) and anti-Dpn (green). EdU was stained for 30 min. EdU signals were detected in *wt* central brain (**H**). Much less EdU signals were detected in *mxc* RNAi central brain (**I**). Scale bars: 20 μm

We quantified the NB numbers in the brain lobes at the 3rd instar larval stage (120 h AEL) for both *wt* and *mxc* knockdown animals (Fig. 3A). In the *wt* brain lobes, the average number of total Dpn^+^ NBs per lobe at 120 h AEL was 72.0 (n = 5 lobes), while in *mxc* knockdown lines, the average number of NBs was reduced to 38.5 (n = 6 lobes; Fig. 3A). In this way it appears that about half of the NBs in *mxc* knockdown brains were missing at the late third-instar larval stage. To exclude the possibility that apoptosis was the cause for NB number changes, *wt* and *mxc* knockdown larval brains were stained with anti-Caspase 3. No obvious signal was identified in either *wt* or *mxc* knockdown NBs (Additional file 1: Fig. S1). The cell sizes of the NBs in the *mxc* RNAi lines had also remained unchanged from those of the *wt* (Additional file 1: Fig. S2).

To determine the time window when the observed premature NB cell fate termination had occurred, we used nuclear Pros as a marker to quantify the ratio of NBs with nuclear Pros at different developmental stages (Fig. 3B). At the second instar larval stage (48 and 72 h AEL) nuclear Pros remained undetected (48 h AEL: 0.71%, n = 239; 72 h AEL: 0.94%, n = 112; Fig. 3B). The nuclear Pros phenotype was first observed at the early third-instar larval stage (96 h AEL; 11.00%, n = 310; Fig. 3B). The average numbers of total NBs per lobe at 96 h AEL was also quantified. The number of NBs in *mxc* RNAi brains was slightly lower than those in the controls (*wt*:71.6, n = 5 lobes; *mxc* RNAi:65.8, n = 5 lobes; Fig. 3A). These data indicate that the NBs in *mxc* knockdown brains begin premature termination at the early third-instar larval stage and shows about half of the NBs missing by the late third-instar larval stage (120 h AEL).

We next performed FLP-out mosaic analysis on *mxc* RNAi lines [34], to evaluate the effects of *mxc* knockdown on the proliferation of NBs. We focused on Type I NB lineages. As shown in Fig. 3C–E, GFP-labeled clonal lineages containing *mxc* knockdown NBs were significantly smaller than those of the *wt* (*wt*: 42.40 cells, n = 5 clones; *mxc* RNAi: 7.18 cells, n = 11 clones). This indicates that, over a given time, cell cycling becomes limited in *mxc* knockdown NBs. In addition, the six-fold cell number difference between the *wt* and the *mxc* knockdown NB lineages also suggests that the cell cycles of GMCs have either become elongated or arrested. To further confirm this, *wt* and *mxc* knockdown larval brains were stained with anti-PH3 (Fig. 3F–G’). No obvious differences of PH3 signals in NBs between *wt* and *mxc* knockdown were observed (Fig. 3F, G). We carried out a standard EdU incorporation experiment (Fig. 3H–I’) and observed far fewer EdU signals in *mxc* knockdown NBs (Fig. 3I), indicating that the lack of Mxc had led to slower rate of cell cycling. These data indicate that Mxc is required for the maintenance of NB cell fate and proliferation in *Drosophila* larval brains.

### Absence of Mxc results in the ectopic expression of Dpn in GMCs

**Fig. 4:**
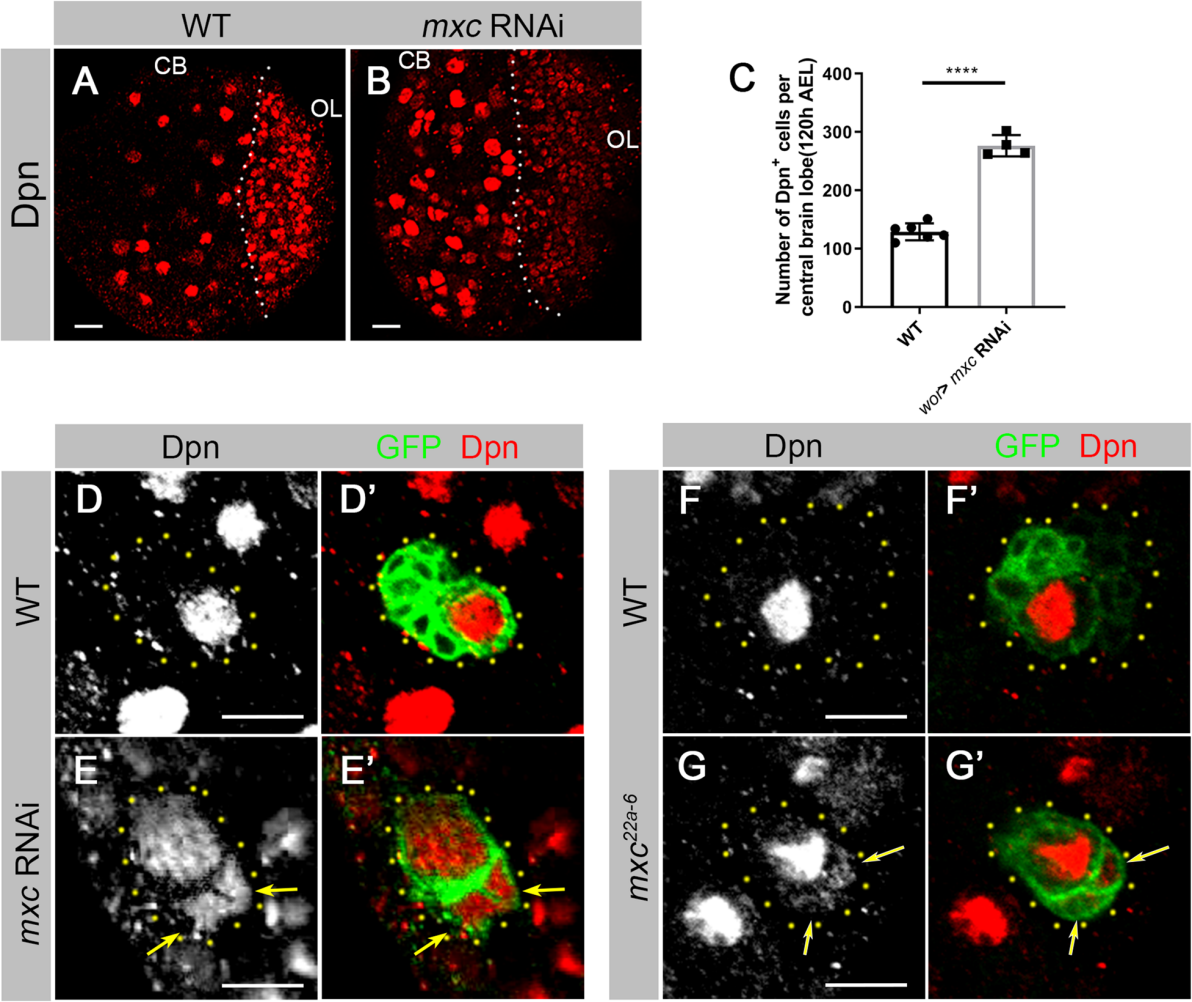
Absence of Mxc results in ectopic expression of Dpn in the progeny of NBs. **A**,** B** Confocal images of the third-instar larval brains of *wt* (**A**) and *mxc* RNAi (**B**), labeled by anti-Dpn (red). Compared to the control (**A**), more Dpn^+^ cells were shown in *mxc* RNAi central brains (**B**). Different cell cycle stages may affect the patterns of Dpn in NB nucleus. (**C**) Statistical analysis of the numbers of Dpn^+^ cells in the larval central brains of *wt* and *mxc* RNAi lines. The data are plotted as mean ± SD. ****p < 0.0001 using a Student’s *t* test, *p* = 5.709E–07. Numbers of brain lobes counted N = 6, N = 4, respectively. **D**–**E’** Third-instar larval brains containing Type-I NB FLP-out clones of *wt* (**D**–**D’**) and *mxc* RNAi (**E**–**E’**), labeled by anti-Dpn (red) and anti-GFP (green). In the *wt* clone (**D**), Dpn was only expressed in the NBs. In the *mxc* RNAi clone (**E**), Dpn was detected in the GMCs (arrowheads). Scale bars: 10 μm. **F**–**G’** Type I NB MARCM clones of *wt* (**F**–**F’**) and *mxc*^*22a − 6*^ (**G**–**G’**) in third-instar larval brains, labeled by anti-Dpn (red) and anti-GFP (green). Dpn (arrowheads) was detected in the GMCs in *mxc*^*22a − 6*^ clone (**G**), but not in *wt* GMCs (**F**). Scale bars: 10 μm

In the central brains of the *wt* larvae, the stem cell marker Dpn is expressed in NBs and mature INPs, but not in GMCs [22, 23, 25]. In addition to normal large sized NBs, we observed many smaller Dpn positive (Dpn^+^) cells in *mxc* knockdown larval brains (Fig. 4A, B). The sizes of these extra Dpn^+^ cells were similar to those of GMCs. To estimate total numbers of Dpn^+^ cells in the central brain, we quantified all Dpn^+^ cells in each brain lobe. An average of 129.0 Dpn^+^ cells/lobe (n = 6 lobes) in *wt* brains and 276.5 Dpn^+^ cells/lobe (n = 4 lobes) in *mxc* knockdown brains were observed (Fig. 4C). This more than two-fold change in Dpn^+^ cell number suggest that more cells are in an undifferentiated state in the absence of Mxc. Since the total number of NBs decreased in *mxc* knockdown brains (Fig. 3A), it is most likely that GMC-like neural precursor cells with Dpn expression contribute to the increased Dpn^+^ populations. As the total number of GMCs is much larger than that of INPs, we focused on the possibility that in the absence of Mxc, large numbers of GMC-like cells, if not all, displayed altered cell fates and ectopically expressed Dpn.

We then established *mxc* RNAi FLP-out clones to analyze the pattern of Dpn in Type I NB lineages (Fig. 4D–E’). In the *wt* Type I NB lineages, the NB was the only Dpn^+^ cell (Fig. 4D). However, as judged by cell sizes, in *mxc* knockdown lineages not just NBs but also GMCs were Dpn positive (Fig. 4E). Similar experiments were carried with genetic mutants (Fig. 4F–G’). In *mxc*^*22a − 6*^ Type I NB MARCM clones, the GMCs were also Dpn^+^ (Fig. 4G). This was identical to the RNAi data (Fig. 4E). These Dpn^+^ GMC-like cells became the major populations of Dpn^+^ cells in the *mxc* knockdown larval brains. We concluded that Mxc acts to suppress the expression of Dpn in GMCs and promotes GMC differentiation.

### The nuclear localization of Pros in NBs and the differentiation of GMCs depend on Mxc-mediated HLB assembly and histone gene transcription

Mxc is a key component of the HLB (Additional file 1: Fig. S3). Using MPM-2 and anti-Mute antibodies, we observed that Mxc was colocalized with another component of HLB, Mute [5, 10, 35, 36], in the nucleus of *wt* NBs, forming unambiguous nuclear foci (arrowhead; Additional file 1: Fig. S4A). The localization of Mute in HLB was Mxc-dependent [5]. In *mxc* knockdown NBs no MPM-2 puncta were detected and the Mute staining pattern was less condensed and with increased numbers of multiple smaller puncta observed compared to the *wt* (Additional file 1: Fig. S4B–B”). Overexpression of Mxc in *mxc* RNAi NBs reversed this phenotype; MPM-2 and Mute restored colocalization at nuclear foci (arrowheads; Additional file 1: Fig. S4C–C”). These data indicate that, in the absence of Mxc, Mute is less condensed and exhibits multiple dot-shaped distributions. Additional experiments were carried out with *mxc*^*16a − 1*^ mutants and *mxc*^*22a − 6*^ MARCM clones. Similar results were observed in both two mutant lines (Additional file 1: Fig. S4D–G”). These results confirm that knockdown *mxc* in larval brain NBs leads to less organized Mute distribution which ultimately impairs HLB assembly.

**Fig. 5:**
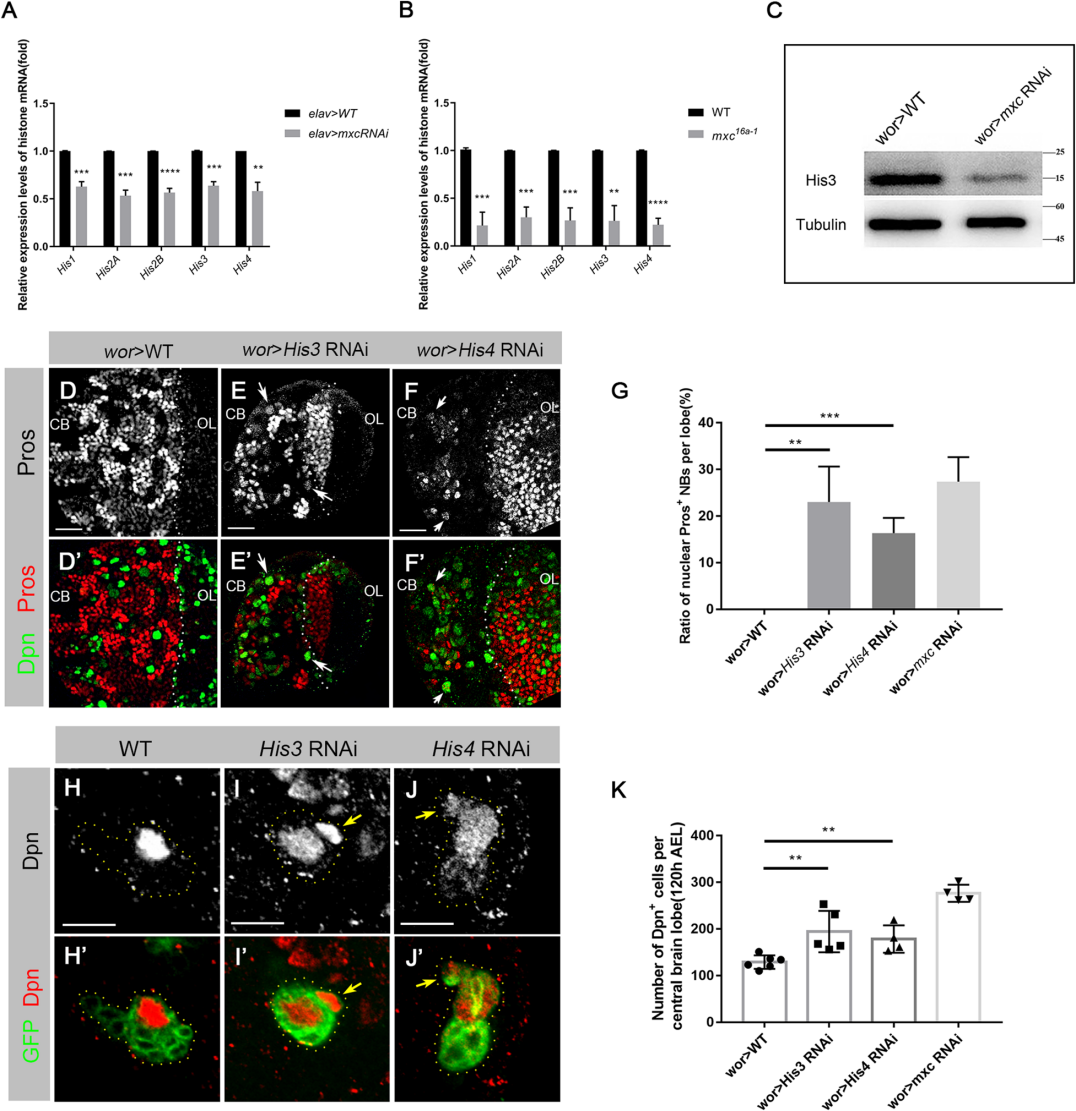
Knocking down ***His3*** or ***His4*** recapitulates the ***mxc*** phenotypes. **A, B** Quantitative RT-PCR results revealed that transcription levels of canonical histone genes were decreased in *mxc* RNAi (**A**) and *mxc*^*16a − 1*^ (**B**) larval brains. The data are plotted as mean ± SD. ****p < 0.0001, ***p < 0.001 and **p < 0.01 using a Student’s *t* test, *p* = 0.0003, 0.0002, 7.758E–05, 0.0001, 0.0014 in (**A**) and 0.0006, 0.0003, 0.0007, 0.0013, 4.019E-05 in (**B**), respectively. Both of the numbers of repetitions N = 3. **C** The western blot results shown that the protein levels of His3 are reduced in *mxc* knockdown larval brain compared to *wt*. **D**–**F’** Third-instar larval brains of *wt* (**D**–**D’**), *His3* RNAi (**E**–**E’**), and *His4* RNAi (**F**–**F’**), labeled by anti-Pros (red) and anti-Dpn (green). Pros (arrowheads) was detected in the nucleus of NBs in both *His3* (**E**) and *His4* (**F**) RNAi lines. Scale bars: 20 μm. **G** Statistical data of nuclear Pros in the NBs in *wt*, *His3* RNAi, *His4* RNAi, and *mxc* RNAi larval central brain. The data are plotted as mean ± SD. **p < 0.01 and ***p < 0.001 using a Student’s *t* test, *p* = 0.0037 and 9.958E-04, respectively. Numbers of NBs counted N = 360, N = 740, N = 151, N = 85, N = 740, respectively. **H**–**J’** Type-I NB FLP-out clones of *wt* (**H**–**H’**), *His3* RNAi (**I**–**I’**) and *His4* RNAi **J**–**J’**, labeled by anti-Dpn (red) and anti-GFP (green). In *wt* clones (H), Dpn was only expressed in the NBs. In both *His3* and *His4* RNAi clones (**I**, **J**), Dpn was detected in the GMCs (arrowheads). Scale bars: 10 μm. **K** Statistical analysis of the numbers of Dpn^+^ cells in the larval central brains of *wt*, *His3* RNAi, *His4* RNAi and *mxc* RNAi lines. The data are plotted as mean ± SD. **p < 0.01 using a Student’s *t* test, *p* = 0.0075 and 0.0070, respectively. Numbers of brain lobes counted N = 6, N = 5, n = 4, N = 4, respectively

We considered whether the absence of Mxc would affect the transcription of histone genes in third-instar larval brains. An *elav*-GAL4 line was used to drive *mxc* RNAi in the larval brains. qPCR analyses showed that the transcription levels of five canonical histone genes were all significantly decreased in both *mxc* knockdown (Fig. 5A) and *mxc*^*16a − 1*^ mutant larval brains (Fig. 5B). Western blot also showed that His3 protein levels had decreased in the *mxc* knockdown larval brains (Fig. 5C), which was consistent with our qPCR results.

To clarify that the reduced histone gene expression is responsible for the *mxc* knockdown-induced NB phenotypes in larval brain, we performed knockdown of either *His3* or *His4* in larval brains and characterized the phenotypes. Nuclear Pros was observed in NBs of both two *His3* RNAi and *His4* RNAi lines (*His3* RNAi:23.03%, n = 151; *His4* RNAi:16.34%, n = 85; Fig. 5D–G), indicating a premature termination of NB cell fate. Quantification of the Dpn^+^ cells in each brain lobe of *His3* RNAi and *His4* RNAi lines showed that averages of 194.4 Dpn^+^ cells (n = 4 lobes) in *His3* knockdown brain lobes and 183.3 Dpn^+^ cells (n = 3 lobes) in *His4* knockdown brain lobes represented significant increases beyond those of the *wt* (129, n = 6 lobes; Fig. 5K). Furthermore, *His3* RNAi and *His4* RNAi FLP-out clones in Type I NB also confirmed that Dpn was ectopically expressed in the GMCs in both *His3* (Fig. 5H-) and *His4* knockdown lineages (Fig. 5J–J’). These data indicate that reduced transcription of histone genes contribute to the blockage of GMC differentiation.

As canonical histones make up the nucleosomes that package the genome to form chromatin [37], a reduction in canonical histone gene transcription of may cause DNA damage and genomic instability. We used anti-phosphorylated H2Av, a marker of DNA double-strand breaks (DSBs) [38–40], to visualize DNA damages in *mxc* knockdown larval brains (Additional file 1: Fig. S5). Obvious phosphorylated H2Av signals were observed (arrowheads; Additional file 1: Fig. S5B), suggesting occurrences of DSBs in the NBs of *mxc* RNAi lines. We also stained the larval brains of *His3* and *His4* RNAi lines with anti-phosphorylated H2Av and observed similarly phosphorylated H2Av signals in these NBs (arrowheads; Additional file 1: Fig. S5C, D). This indicates that knockdown of *His3* or *His4* recapitulates the *mxc* phenotypes. Taken together, these results demonstrate that Mxc-mediated HLB assembly and histone gene transcription are critical for the maintenance of NBs cell fate and their progeny in larval brains.

### The increase in autophagy induced by mxc knockdown as a defense mechanism in NBs

**Fig. 6:**
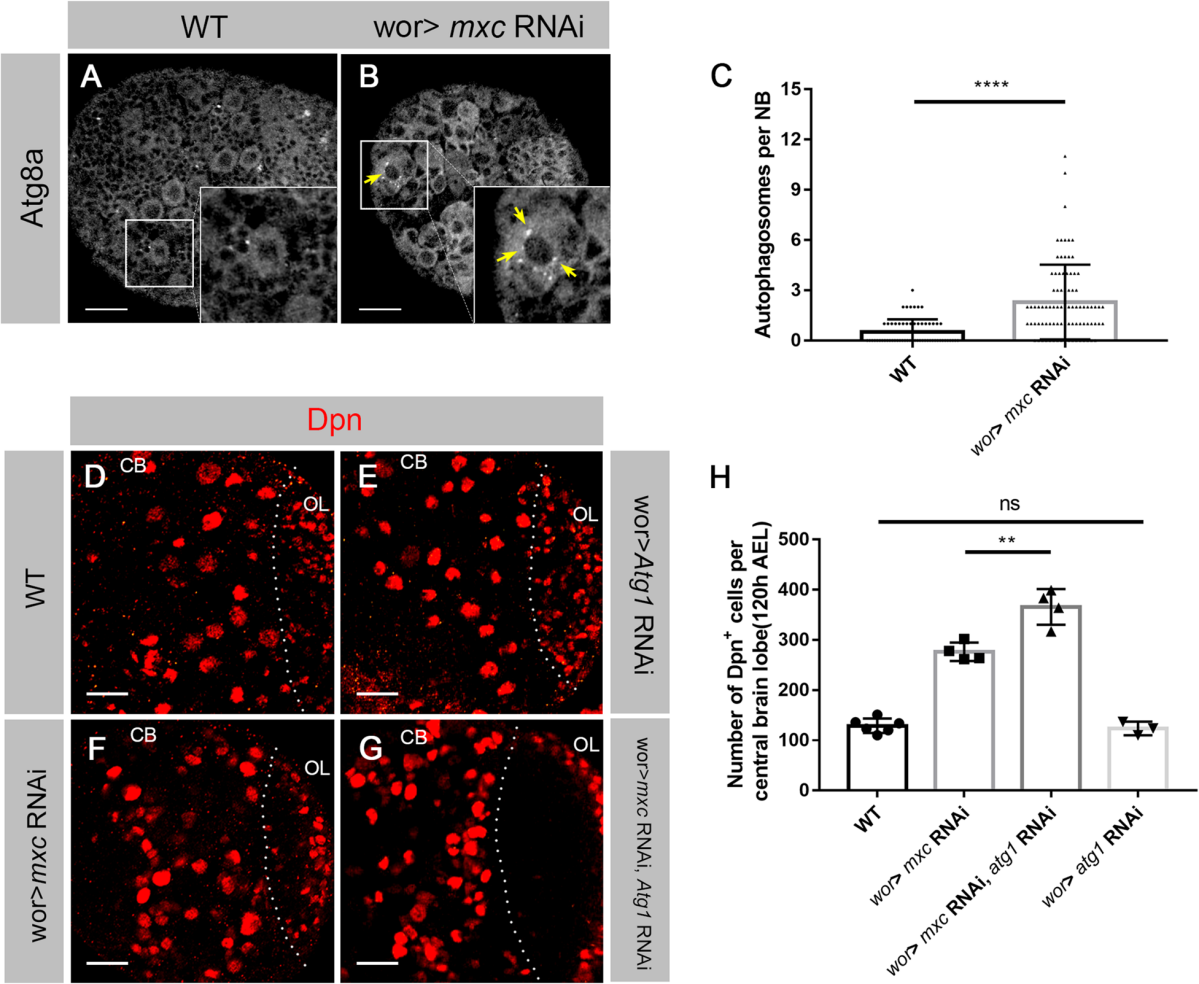
Autophagy was activated to inhibit the production of Dpn ^+^ GMCs in ***mxc*** knockdown NBs. **A**–**B’** Third-instar larval brains of *wt* (**A**–**A’**) and *mxc* RNAi (**B**–**B’**), labeled by anti-Dpn (red) and anti-Atg8a (green). In *wt* larval central brain (**A**), only basic signals of Atg8a were detected. In *mxc* RNAi larval central brain (**B**), an increased number of Atg8a puncta (arrowheads) was detected in the cytoplasm of the NB. Scale bars: 20 μm. **C** Statistical analysis of autophagy levels in the NBs of *wt* and *mxc* RNAi larval central brains. The data are plotted as mean ± SD. ****p < 0.0001 using a Student’s *t* test, *p* = 1.764E-08. Numbers of NBs counted N = 60, N = 87, respectively. **D**–**G** Third-instar larval brains of *wt* (**D**), *Atg1* RNAi (**E**), *mxc* RNAi (F) and *Atg1* RNAi, *mxc* RNAi double knockdown (**G**) lines, labeled by anti-Dpn (red). *Atg1* RNAi (**E**) larval brains showed the same phenotype as the *wt* (**D**). Central brains in *Atg1* RNAi, *mxc* RNAi double knockdown lines (**G**) showed more Dpn^+^ cells than those in *mxc* RNAi lines (**F**). Scale bars: 20 μm. (**H**) Statistical data of the numbers of Dpn^+^ cells in the larval central brains of *wt*, *mxc* RNAi, *Atg1* RNAi and *Atg1* RNAi, *mxc* RNAi double knockdown lines. The data are plotted as mean ± SD. **p < 0.01 and ns not significant using a Student’s *t* test, *p* = 0.0043 and 0.6104, respectively. Numbers of brain lobes counted N = 6, N = 4, N = 4, N = 3, respectively

Persistent DNA DSBs can represent genotoxic stress on a cellular level, in which the DNA damage response (DDR) pathway is activated to combat this, which induces a variety of biological processes [41]. Autophagy, a lysosome-dependent degradation pathway, is one of the processes activated via the DDR pathway [42, 43]. According to previous reports, autophagy is thought to have dual roles in responding to DNA damage [44–48]. To explore whether autophagy is activated in *mxc* knockdown NBs, we stained *wt* and *mxc* knockdown larval brains with anti-Atg8a, a marker of autophagosomes [49]. In *wt* NBs, only a basal Atg8a signal was detected (Fig. 6A), suggesting autophagy only occurred at a low level. In contrast, an increased signal of Atg8a puncta was observed in the cytoplasm of *mxc* knockdown NBs (arrowheads; Fig. 6B), indicating an accumulation of autophagosomes. We quantified the Atg8a puncta in *wt* and *mxc* knockdown NBs (Fig. 6C). The number of dots per NB in *mxc* knockdown lines (2.30; n = 87) was significantly higher than that in the *wt* NBs (0.52; n = 60). To further verify the activation of autophagy, exogenous ATG8a-GFP was expressed as a visual reporter of autophagosome in NBs (Additional file 1: Fig. S6D, E). ATG8a-GFP puncta were clearly observed as accumulated in the cytoplasm of *mxc* knockdown NBs (arrowheads; Additional file 1: Fig. S6E), but with almost no obvious corresponding signals in *wt* NBs (Additional file 1: Fig. S6D). Similar experiments were carried out with *mxc*^*16a − 1*^ mutants (Additional file 1: Fig. S6A, B). The number of ATG8a-GFP dots per NB in *mxc*^*16a − 1*^ mutants (3.48; n = 50) were also significantly higher than that those of *wt* NBs (0.33; n = 57; Additional file 1: Fig. S6C). These results were consistent with *mxc* knockdown data, indicating that *mxc* deficiency triggers an increased autophagy in NBs.

We performed knockdown of *Atg1*, a key gene of the autophagy pathway [50, 51], in the NBs of both *wt* and *mxc* knockdown lines (Fig. 6D–H). No obvious phenotype was observed in *Atg1* knockdown *wt* larval brains (Fig. 6E, H), which was consistent with the low levels of autophagy detected above (Fig. 6A). In *Atg1* and *mxc* double knockdown larval brains, more GMC-like Dpn^+^ cells were detected, as compared with that in only *mxc* knockdown larval brains (Fig. 6F, G). Quantification showed a higher number with about 368 Dpn^+^ cells/lobe (n = 4 lobes) in double knockdown brain lobes, as compared with that in *mxc* knockdown brains, with about 276.5 Dpn^+^ cells/lobe (n = 4 lobes; Fig. 6H). These data suggest that elevated autophagy induced by Mxc deficiency in NBs might be a defense mechanism responding to abnormal HLB assembly and consequently pre-terminated NB cell fate.

## Discussion

NPAT/Mxc is a component of the HLB, participating in transcription of canonical histone genes and in pre-mRNA processing [5, 10]. Although *NPAT* gene has been reported as a candidate gene for mental retardation, no genetic mutations of *NPAT* gene have been found in clinic cases. Our study shows that knockdown *mxc* in larval brain NBs causes the reduction of transcription levels of histone genes and increased DNA DSBs. These defects lead to premature termination of NB cell fate and blockage of GMC differentiation processes, resulting in neuronal and locomotion defects in adult flies.

In *mxc* knockdown flies, total NB numbers decrease and its proliferation potential is disrupted, leading to severe neuronal loss in the adult central brain. The mutant flies show locomotion disorder and survive no more than two days after eclosion. It is notable that the hemizygotes of *mxc*^*16a − 1*^ die as late larvae or early pupae. NB specific overexpression of Mxc in *mxc*^*16a − 1*^ mutants rescues these animals, at least to the late pupal stage. Some of these flies then go on to survive to adulthood without obvious mobility problems. This indicates that *mxc* mutant lethality at larval or early pupal stage is mainly due to the disruption of CNS development. Such results support the hypothesis that *NPAT/mxc* deficiency could act as a pathogenic factor for neurodevelopmental disorders such as mental retardation.

Notably the hemizygotes of *mxc*^*16a − 1*^ exhibits weaker phenotype of nuclear Pros in NBs and die as late larvae or early pupae. Since *mxc*^*16a − 1*^ mutation only occurred from the 1824 aa at the very C-terminal end with the rest aa sequence unchanged, it is likely that the very C-terminal sequence is required for colocalization of MPM2 and Mute in NBs while some other domain(s) resided within unchanged N-terminal region maintain(s) function to prevent nuclear Pros formation.

It has been reported that the ectopic expression of Dpn in immature INP cells can transform these cells into NBs-like cells that divide uncontrollably, causing tumor formation [23]. Although Dpn^+^ GMCs might have proliferation potential, no tumor over-growth phenotype is observed in our study. This may reflect the fact that in addition to NBs, the cell-cycles of GMCs in *mxc* knockdown lines are also slowed down or even arrested. The slowed down cell cycle seems to protect NBs and GMCs from tumor over-growth. We propose that in *wt* NBs, Mxc acts to suppress Dpn expression in GMCs and promotes GMC differentiation. This conclusion is consistent with notion that in the absence of Mxc functions, large populations of GMC-like cells continue to express Dpn, and their differentiation process is blocked. The results from *His3* and *His4* RNAi lines also show Dpn^+^ GMCs phenotypes consistent to the *mxc* data.

DSBs are often considered to be a factor for tumor formation [52]. A previous report demonstrated that *mxc* knockdown and *mxc*^*16a − 1*^ led to larval lymph gland hyperplasia [14]. Interestingly, in our study, although knockdown of *mxc* or histone genes cause severe DSBs in the larval brains, there was no evidence suggesting the over-growth of NBs or GMCs. Such a discrepancy of Mxc function between lymph gland cells [14] and NBs could possibly be due to the cell fate determinant, Pros. The premature accumulation of nuclear Pros in larval brain NBs is likely to be a mechanism to prevent NBs from over-proliferation when under a state of DSBs stress or perhaps under other instances of genomic instability. Similar results have been reported in that ionizing radiation treatments are noted to induce both DSBs and prematurely accumulated nuclear Pros in *Drosophila* larval brain NBs [53], with no NB over-proliferation observed. Premature nuclear Pros, that early terminates the NB cell fate and diminishes their proliferative properties, may play a role as a protective mechanism to avoid NB over-growth.

Autophagy is a conserved cellular process which can be induced under stress conditions such as nutrient starvation or DNA damage [43, 54]. At present, no data has been presented describing the role of autophagy in the larval brain NB development of *Drosophila*. Based on our data, autophagy shows a protective role against Dpn^+^ GMC formation. In the *wt* larval brain NBs, autophagy is kept at a low level. However, the autophagy levels are elevated in *mxc* knockdown NBs. Blocking the autophagy process by knocking down *Atg1* enhances the Dpn^+^ GMC population. Although autophagy does not appear to be required in *wt* larval brain NBs under normal conditions, it represents a protective function under the DSBs stress induced by *mxc* knockdown.

## Conclusions

**Fig. 7:**
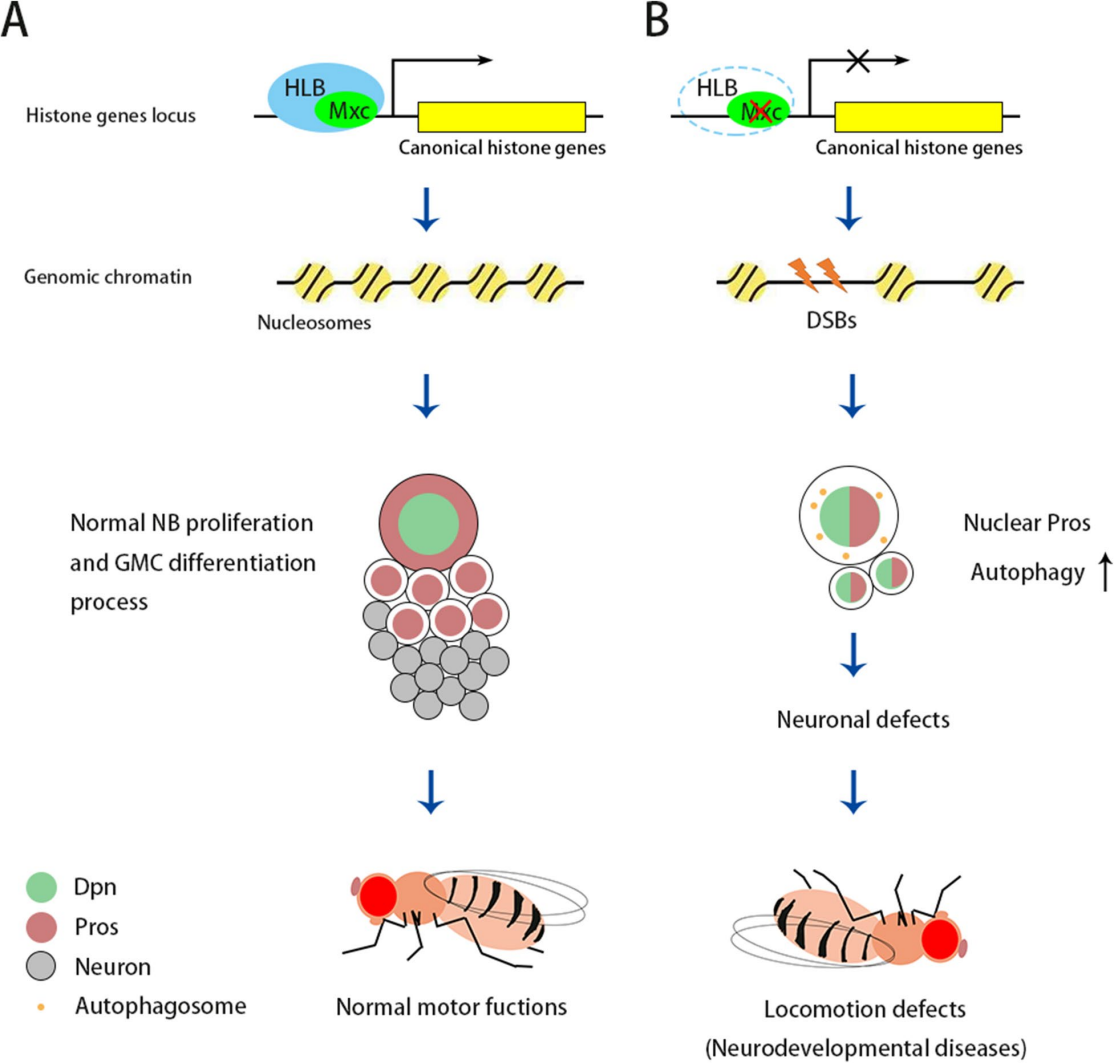
Diagram depicting the mechanisms of neuronal and locomotion defects caused by *mxc* knockdown in *Drosophila* larval brain NBs. **A** In *wt*, Mxc is required for HLB assembly and histone transcription, which maintains genomic stability and CNS development. **B** The absence of Mxc impairs HLB assembly which in turn reduces the transcription levels of canonical histone genes and induces DSBs. NB proliferation and GMC differentiation is affected under this stress, resulting in neuronal and locomotion defects in adult flies. Nuclear Pros is prematurely accumulated in third-instar larval brain NBs to terminate NB cell fate, potentially protecting from NB over-growth. Autophagy is also elevated in *mxc* knockdown NBs to impede Dpn^+^ GMC formation

Our study revealed the functions of Mxc in the CNS development (Fig. 7). The lack of canonical histone genes caused by *mxc* knockdown leads to lower NB proliferation potential and premature termination of NB cell fate, as well as blockage of differentiation process of GMCs, which ultimately results in neuronal defects in adult flies. Our study provides the first important clue that *NPAT*/*mxc* may act as a pathogenic factor for neurological disease, potentially those related to mental retardation.

## Materials and methods

### Fly stocks and genetics

All flies were maintained at 25℃. The *Drosophila* stocks (and their sources), used in this study include: *w*^*1118*^, UAS-*Dicer2*; *wor*-GAL4, *mxc*^*RNAi*^ (THU0893 and THU5844, Tsinghua *Drosophila* center), *mxc*^*16a − 1*^ (7133, BDSC), *mxc*^*22a − 6*^ (7128, BDSC), *His3*^*RNAi*^ (THU4319, Tsinghua *Drosophila* center), *His4*^*RNAi*^ (109,060, VDRC), *Atg1*^*RNA*i^ (THU2357, Tsinghua *Drosophila* center), *elav*-GAL4, UAS-Atg8a-GFP (51,656, BDSC).

UAS-Mxc was generated in this study. To generate *pUAST-mxc* construct, the full‐length *mxc* cDNA was amplified with the primers 5′‐CGGAATTCATGGAGTCGATTGTCCTGCATTC‐3′ and 5′‐TAAAGCGGCCGCTCAGGTGCCGTGCAGATGTGA‐3′. The sequence was cloned into a *pUAST* vector. The construct was injected into the VK33 lines at the Core Facility of *Drosophila* Resource and Technology, CEMCS, CAS, following standard methods.

MARCM analysis were performed as previously described [33, 55]. For *mxc*^*22a − 6*^ clones, we used the fly line with the genotype of *hsFLP*, *P{FRT}19 A tub*-GAL80/FM6; *wor*-GAL4, UAS-CD8-GFP/*CyO* as the tool. *P{FRT}19 A mxc*^*22a − 6*^/FM6 was obtained by homologous recombination of lines *P{FRT}19 A* (1744, BDSC) and *mxc*^*22a − 6*^/FM7c (7128, BDSC). The MARCM clones were GFP labeled.

For FLP-out mosaic analysis, the fly line with the genotype of hsFLP; *act* > *y*^*+*^>GAL4, UAS-CD8-GFP was used to cross with RNAi lines [34]. The FLP-out clones were labeled with GFP. Both MARCM clones and RNAi FLP-out clones were induced using a 45 min heat-shock treatment of 37 °C at 32–36 h after egg laying.

### Antibody generation

The sequence of Mute for the antibody, aa 3-164, was selected according to Bulchand’s work [36]. The sequence was cloned into a *pGEX-4T-1* expression vector. The antigen was bacterially produced and the antibody was raised in guinea pigs.

### Immunofluorescence staining and antibodies

Different stages of larval brains and adult brains were dissected in ice-cold Schneider’s *Drosophila* Medium (Gibco). Samples were fixed for 18 min in PBS (10 mM NaH2PO4/Na2HPO4, 175 mM NaCl, pH7.4) with 4% paraformaldehyde at room temperature [55, 56]. The samples were incubated with primary antibodies at 4℃ overnight, and then with secondary antibodies at room temperature for 1–2 h. Antifade Mounting Medium (P0126, Beyotime, China) was used to protect the fluorescent signals of the samples. The images were obtained using an Olympus FV1000 confocal microscope and processed using Adobe Photoshop CC 2018.

The primary antibodies in this study were as follows: mouse anti-Pros (1:50, DSHB); guinea pig anti-Dpn (1:1000, a gift from Y. Cai); rabbit anti-Ase (1:1000, a gift from Y. Cai); chicken anti-GFP (1:1000, ab13970, Abcam); rabbit anti-Mute (1:1000, this study); mouse MPM-2 (1:1000, 05-368MG, Sigma-Aldrich); rabbit anti-Caspase 3 (1:1000, Asp175, Cell Signaling); rabbit anti-Phospho-Histone3 (Ser10) (1:1000, Millipore); rat anti-Elav (1:50, DSHB); rabbit anti-Histone H2AvD pS137 (1:1000, Rockland); rabbit anti-GABARAP + GABARAPL1 + GABARAPL2, used as anti-Atg8a in *Drosophila* (1:200, ab109364, Abcam) [49]. All commercial secondary antibodies used were from the Jackson Laboratory. DNA was stained with DAPI (C1002, Beyotime) at 1:2000. For EdU analysis, samples were incubated for 30 min in 5 mM EdU before fixing, using Click-iT EdU Imaging Kits (Alexa Fluor 555) (C10338, Invitrogen).

### Western blot

Third-instar larval brains were lysed in RIPA lysis buffer [50 mM Tris-HCl pH 8.0, 150 mM NaCl, 0.5% sodium deoxycholate, 0.1% SDS, 1% IGEPAL CA-630 and complete protease inhibitor cocktail (Roche)]. About 30 brains were lysed in each sample. Samples were subjected to SDS‐PAGE and transferred to a polyvinylidene fluoride membrane. Mouse anti-His3 (1:1000; Active Motif) and mouse anti-β-Tubulin (1:1000; DSHB) were used as primary antibodies, and HRP, anti‐mouse (1:5000; Abcam) were used as secondary antibodies.

### Quantitative RT-PCR

RNA was extracted by the TRIzol (Invitrogen) method. About 30 brains of third-instar larvae were dissected for each sample. HiScript III 1st Strand cDNA Synthesis Kit (Vazyme, China) was used for reverse transcription according to the manufacturer’s protocol. Note that it was necessary to select Random hexamers rather than Oligo (dT) for reverse transcription of histone mRNA.

7900HT Fast Real-Time PCR System (Applied Biosystems) was used for quantitative RT-PCR. PCR was carried out in the system with Power SYBR Green PCR Mix and Dye I (Vazyme) in 96-well plates. Each well contained 1 µl of the retro-transcription product, 0.25 µl Dye I and 0.5 µl forward and reverse primers (6.25µM) in 1× SYBR Green Mix, in a final volume of 12.5 µl. *rp49* was used as an internal control. The program was: 50 °C for 2 min, 95 °C denaturation for 10 min, followed by 40 amplification cycles (15 s at 95 °C, 60 s at 60 °C), and ended by a melting curve (15 s at 95 °C, 60 s at 60 °C, 30 s at 95 °C and 15 s at 60 °C). Forward and reverse primers were used in this paper: For *rp49*, forward: 5′ GCTAAGCTGTCGCACAAA, reverse: 5′ TCCGGTGGGCAGCATGTG; for *His3*, forward: 5′ ACCGAGCTTCTAATCCGCAAG, reverse: 5′ ACCAACCAGGTAGGCTTCGC; for *His4*, forward: 5′ TGGCGTTCTGAAGGTTTTCTTG, reverse: 5′ AACCGCCAAATCCGTAGAGG; for *H2a*, forward: 5′ GAATTATTCCGCGTCATCTGC, reverse: 5′ TTCTCGGTCTTCTTGGGCAAC; for *H2b*, forward: 5′ CGTCGAAGGCGATGAGCATAA, reverse: 5′ AGGCGAACAGCCGTTTGGATC; for *H1*, forward: 5′ GCCGAAAATAAGAAAACTGA, reverse: 5′ CTTTGACGCTTTCGCTACTA.

### Statistical analysis

All data are expressed as the mean ± SD. All statistical data are processed using unpaired two-tailed Student’s *t*-test in GraphPad Prism.

## Supplementary Information

**Additional file 1**: **Figure S1.** Knockingdown mxc does not induce apoptosis. (A-B’) Third‐instar larval brains of wt (A-A’) and mxc RNAi (B-B’), labeled by anti‐Dpn (red) and anti‐Caspase3(green). Only background Caspase3 signals were detected in the *wt* (A) and *mxc* RNAi (B) larvalbrains. Scale bars: 20 μm. **Figure S2. **No cell size change in mxc RNAi NBs.Statistical data of cell size of the NB in wt and mxc RNAi third-instar larval brains. The data areplotted as mean ± SD. No significant difference using a Student’s *t*test, *p* = 0.3439. Both of the numbers of neuroblasts counted N = 15. **FigureS3.** Schematic of the components of Histone locus body (HLB). The HLBis formed via a hierarchical recruitment of components. Mxc and FLASH are thecore components of HLB formation. Mute, Slbp, U7 snRNP and other components arerecruited by Mxc and FLASH for canonical histone gene transcription and pre-RNAprocessing ^5,16^. **Figure S4. **Absence of mxc impairs HLBformation in NBs. (A-C”) Third‐instarlarval NBs of *wt* (A-A”), *mxc*RNAi (B-B”), and *mxc* RNAi with UAS-Mxc (C-C”) labeled by anti‐Mute (red), MPM2 antibody (green) and Dapi(blue). In *wt* NBs (A-A”), MPM2signals and Mute signals were colocalized and formed nuclear foci (arrowheads).In *mxc* RNAi NBs (B-B”), no MPM2 puncta were detected (B’). The Mute patterns (arrowheads) wereless condensed and multiple smaller puncta were observed in the NBs (B”). Overexpression of Mxc in *mxc*RNAi background (C-C”) rescued the phenotype, MPM2 and Mute were colocalized atthe nuclear foci (arrowheads). Scale bars: 10 μm. (D-E”) Third‐instarlarval NBs of wt (D-D”) and mxc^16a-1^(E-E”), labeled by anti‐Mute(red), MPM2 antibody (green) and Dapi (blue). Multiple smaller puncta were observed in mxc^16a-1^NBs and these MPM2 and Mute signals were not colocalized (E-E”). Scale bars: 10μm. (F-G”) MARCM clones of wt (F-F”) and mxc^22a-6^ (G-G”)in third‐instar larval brains, labeled by MPM2 (red),anti-Mute (blue) and anti-GFP (green). In mxc^22a-6^NBs, noMPM2 puncta were detected(G’), and Mute patterns were also shown as multiple puncta (G”). Scale bars: 10μm. **Figure S5. ** Knocking down mxc or histone genes leads to DNA DSBs. (A-D’)Confocal images of the third‐instarlarval brains of *wt* (A-A’), *mxc*RNAi (B-B’), *His3* RNAi (C-C’) and *His4* RNAi (D-D’), labeled byanti‐H2Av pS137 (red) and anti‐Dpn (green). In *wt* larval central brain (A), no obviously phosphorylated H2Avsignals were detected. Strong signals (arrowheads) were detected in the centralbrains of *mxc* RNAi (B), *His3* RNAi (C) and *His4* RNAi (D)lines. Scale bars: 20 μm. **Figure S6. **Autophagy was activated in mxcknockdown or mutant NBs. (A-B) Third‐instarlarval brains of *wt* (A) and* mxc*^16a-1^(B), labeled by anti‐Dpn (red) and anti‐GFP (green). These lines contained UAS-Atg8a-GFPdriven by *wor*-GAL4. In the *wt*larval central brain (A), only the basic signal of Atg8a-GFP was detected. Inthe *mxc*^16a-1^ larval central brain (B), obvious Atg8a puncta(arrowheads) were detected in the cytoplasm of the NB. Scale bars: 20 μm. (C) Statistical analysis of autophagy levelsin the NBs of *wt* and *mxc*^16a-1^larval central brains. The data are plotted as mean ± SD. ****p <0.0001 using a Student’s *t* test, *p* = 7.441E-11. Numbers of NBscounted N = 57, N = 50, respectively.(D-E) Third‐instar larval brains of *wt* (D) and* mxc* RNAi (E), labeledby anti‐Dpn (red) and anti‐GFP(green). These lines contained UAS-Atg8a-GFP which was driven by *wor*-GAL4.Obvious Atg8a-GFP puncta (arrowheads) were also detected in the cytoplasm of *mxc*knockdown NB. Scale bars: 20 μm.

## Data Availability

Data sharing is not applicable to this article as no datasets were generated or analyzed during the current study. The manuscript has data included as electronic supplementary material.
