# Supplementary material for: Mxc, a Drosophila homolog of mental retardation-associated gene NPAT, maintains neural stem cell fate
Source: Cell Biosci. 2022 May 31;12:78. doi: 10.1186/s13578-022-00820-8 (PMC9153134; doi:10.1186/s13578-022-00820-8)
Supplement: Supplementary file 1 — Additional file 1: Figure S1. Knockingdown mxc does not induce apoptosis. (A-B’) Third‐instar larval brains of wt (A-A’) and mxc RNAi (B-B’), labeled by anti‐Dpn (red) and anti‐Caspase3(green). Only background Caspase3 signals were detected in the wt (A) and mxc RNAi (B) larvalbrains. Scale bars: 20 μm. Figure S2. No cell size change in mxc RNAi NBs.Statistical data of cell size of the NB in wt and mxc RNAi third-instar larval brains. The data areplotted as mean ± SD. No significant difference using a Student’s ttest, p = 0.3439. Both of the numbers of neuroblasts counted N = 15. FigureS3. Schematic of the components of Histone locus body (HLB). The HLBis formed via a hierarchical recruitment of components. Mxc and FLASH are thecore components of HLB formation. Mute, Slbp, U7 snRNP and other components arerecruited by Mxc and FLASH for canonical histone gene transcription and pre-RNAprocessing 5,16. Figure S4. Absence of mxc impairs HLBformation in NBs. (A-C”) Third‐instarlarval NBs of wt (A-A”), mxcRNAi (B-B”), and mxc RNAi with UAS-Mxc (C-C”) labeled by anti‐Mute (red), MPM2 antibody (green) and Dapi(blue). In wt NBs (A-A”), MPM2signals and Mute signals were colocalized and formed nuclear foci (arrowheads).In mxc RNAi NBs (B-B”), no MPM2 puncta were detected (B’). The Mute patterns (arrowheads) wereless condensed and multiple smaller puncta were observed in the NBs (B”). Overexpression of Mxc in mxcRNAi background (C-C”) rescued the phenotype, MPM2 and Mute were colocalized atthe nuclear foci (arrowheads). Scale bars: 10 μm. (D-E”) Third‐instarlarval NBs of wt (D-D”) and mxc16a-1(E-E”), labeled by anti‐Mute(red), MPM2 antibody (green) and Dapi (blue). Multiple smaller puncta were observed in mxc16a-1NBs and these MPM2 and Mute signals were not colocalized (E-E”). Scale bars: 10μm. (F-G”) MARCM clones of wt (F-F”) and mxc22a-6 (G-G”)in third‐instar larval brains, labeled by MPM2 (red),anti-Mute (blue) and anti-GFP (green). In mxc22a-6NBs, noMPM2 puncta were detecte [file 13578_2022_820_MOESM1_ESM.docx]

## Mxc, a *Drosophila* homolog of mental retardation-associated gene *NPAT*, maintains neural stem cell fate

Rong Sang ^1, 2,^ ^#^, Cheng Wu ^1, #^, Shanshan Xie ^1^, Xiao Xu ^1^, Yuhan Lou ^1^, Wanzhong Ge ^1^, Yongmei Xi ^1, *^, Xiaohang Yang ^1, 3, *^

## Additional file


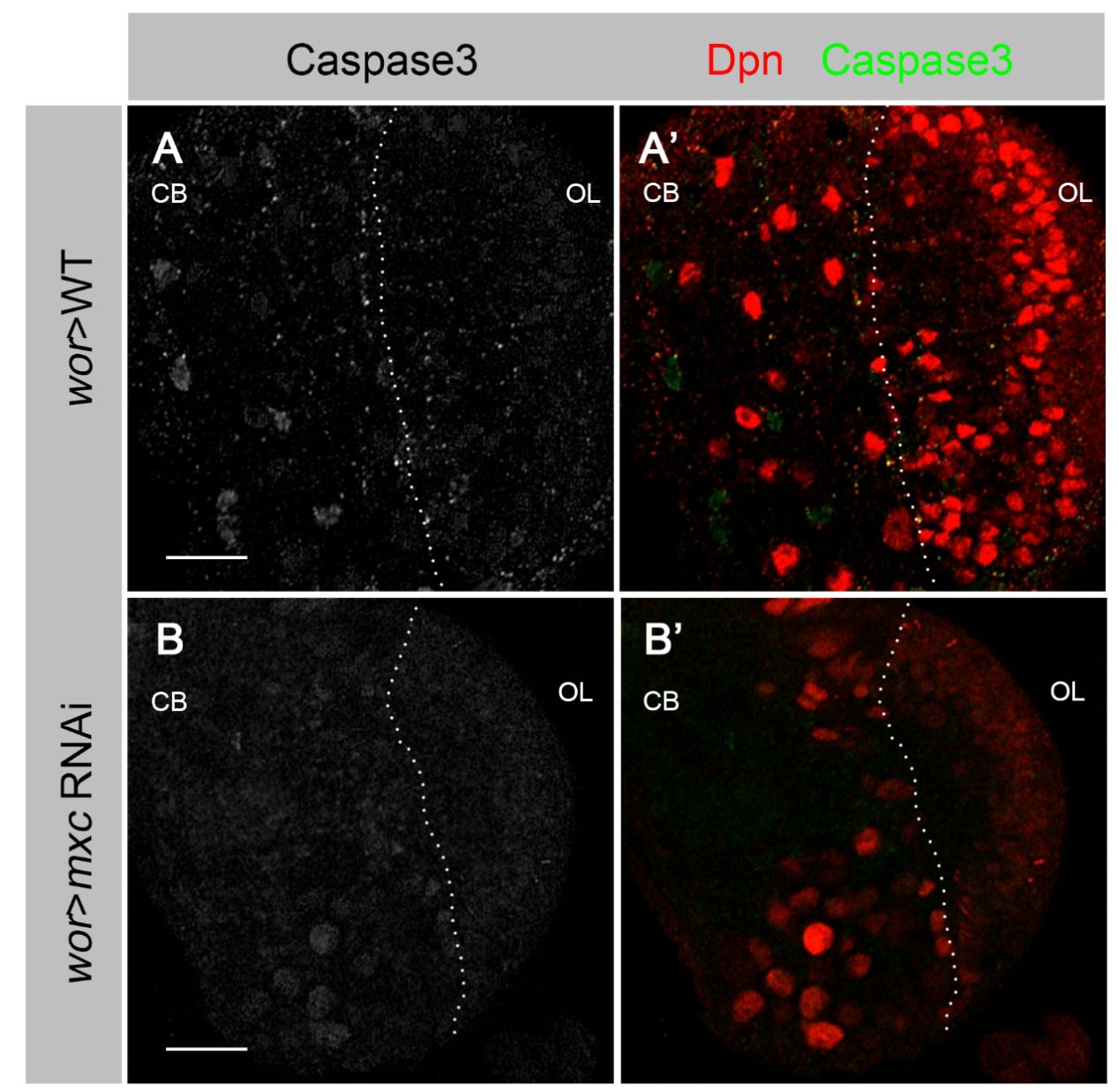


**Figure S1** **Knocking down *mxc* does not induce apoptosis.**

(A-B’) Third‐instar larval brains of *wt* (A-A’) and *mxc* RNAi (B-B’), labeled by anti‐Dpn (red) and anti‐Caspase3 (green). Only background Caspase3 signals were detected in the *wt* (A) and *mxc* RNAi (B) larval brains. Scale bars: 20 μm.


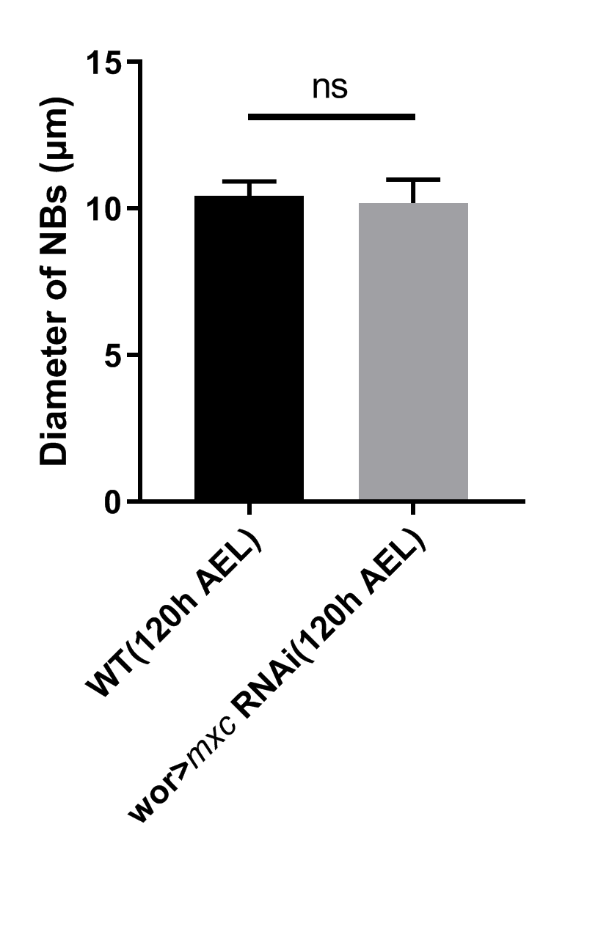


**Figure S2 No cell size change in *mxc* RNAi NBs.**

Statistical data of cell size of the NB in *wt* and *mxc* RNAi third-instar larval brains. The data are plotted as mean ± SD. No significant difference using a Student’s *t* test, *p* = 0.3439. Both of the numbers of neuroblasts counted N = 15.


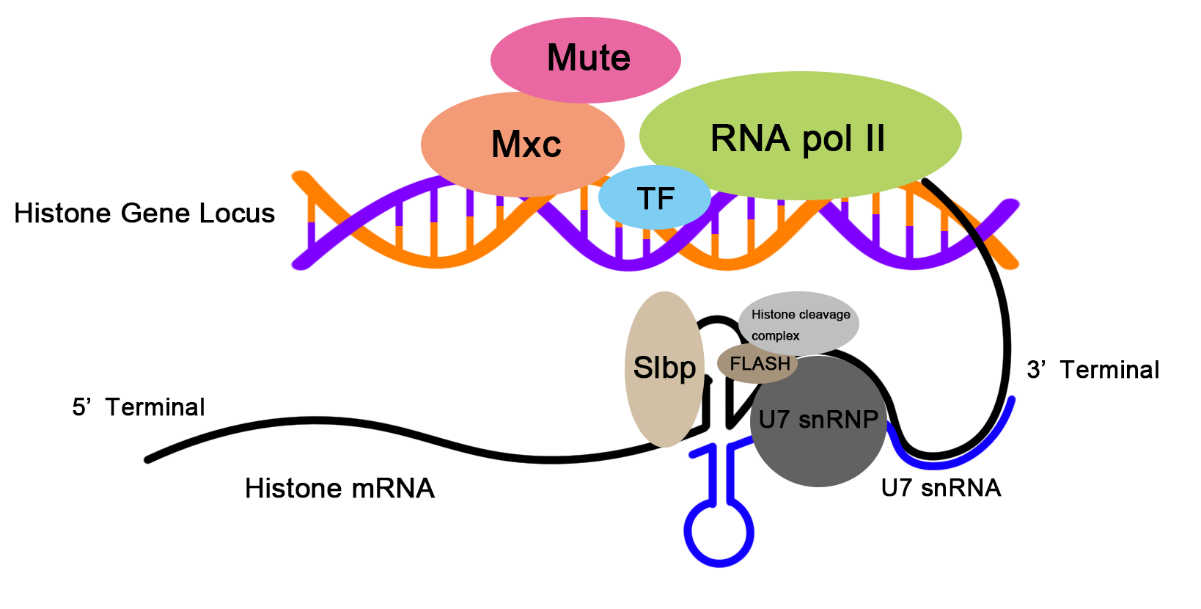


**Figure S3** **Schematic of the components of Histone locus body (HLB).**

The HLB is formed via a hierarchical recruitment of components. Mxc and FLASH are the core components of HLB formation. Mute, Slbp, U7 snRNP and other components are recruited by Mxc and FLASH for canonical histone gene transcription and pre-RNA processing ^5,16^.


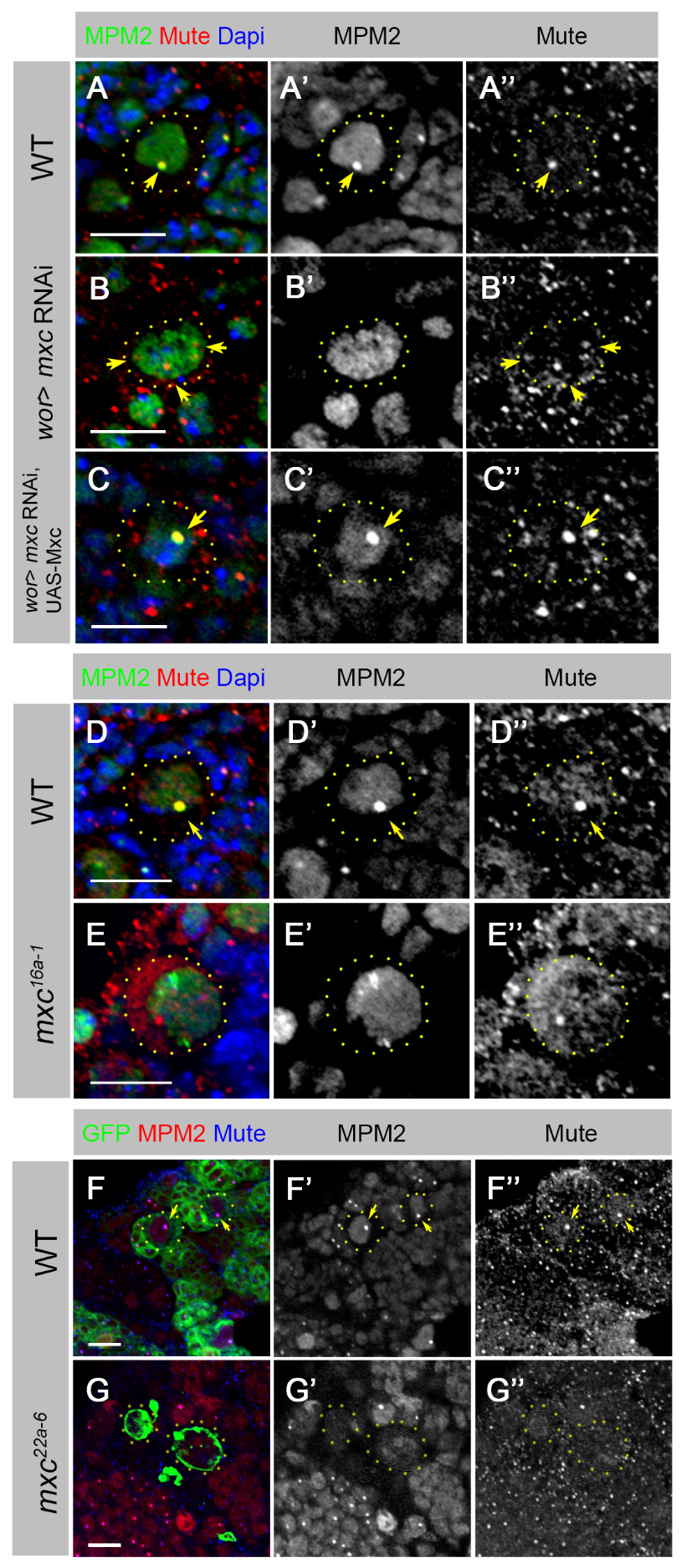


**Figure S4 Absence of Mxc impairs HLB formation in NBs.**

(A-C”) Third‐instar larval NBs of *wt* (A-A”), *mxc* RNAi (B-B”), and *mxc* RNAi with UAS-Mxc (C-C”) labeled by anti‐Mute (red), MPM2 antibody (green) and Dapi (blue). In *wt* NBs (A-A”), MPM2 signals and Mute signals were colocalized and formed nuclear foci (arrowheads). In *mxc* RNAi NBs (B-B”), no MPM2 puncta were detected (B’). The Mute patterns (arrowheads) were less condensed and multiple smaller puncta were observed in the NBs (B”). Overexpression of Mxc in *mxc* RNAi background (C-C”) rescued the phenotype, MPM2 and Mute were colocalized at the nuclear foci (arrowheads). Scale bars: 10 μm. (D-E”) Third‐instar larval NBs of *wt* (D-D”) and *mxc^16a-1^* (E-E”), labeled by anti‐Mute (red), MPM2 antibody (green) and Dapi (blue). Multiple smaller puncta were observed in *mxc^16a-1^* NBs and these MPM2 and Mute signals were not colocalized (E-E”). Scale bars: 10 μm. (F-G”) MARCM clones of *wt* (F-F”) and *mxc^22a-6^* (G-G”) in third‐instar larval brains, labeled by MPM2 (red), anti-Mute (blue) and anti-GFP (green). In *mxc^22a-6^* NBs, no MPM2 puncta were detected (G’), and Mute patterns were also shown as multiple puncta (G”). Scale bars: 10 μm.


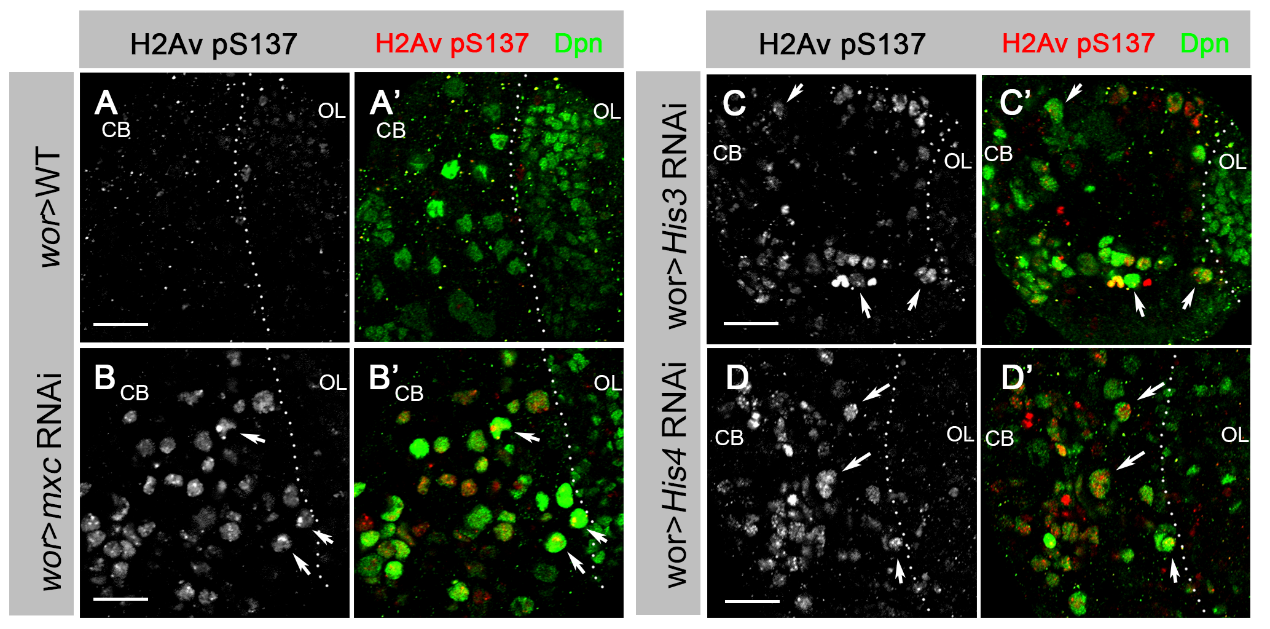


**Figure S5 Knocking down *mxc* or histone genes leads to DNA DSBs.**

(A-D’) Confocal images of the third‐instar larval brains of *wt* (A-A’), *mxc* RNAi (B-B’), *His3* RNAi (C-C’) and *His4* RNAi (D-D’), labeled by anti‐H2Av pS137 (red) and anti‐Dpn (green). In *wt* larval central brain (A), no obviously phosphorylated H2Av signals were detected. Strong signals (arrowheads) were detected in the central brains of *mxc* RNAi (B), *His3* RNAi (C) and *His4* RNAi (D) lines. Scale bars: 20 μm.


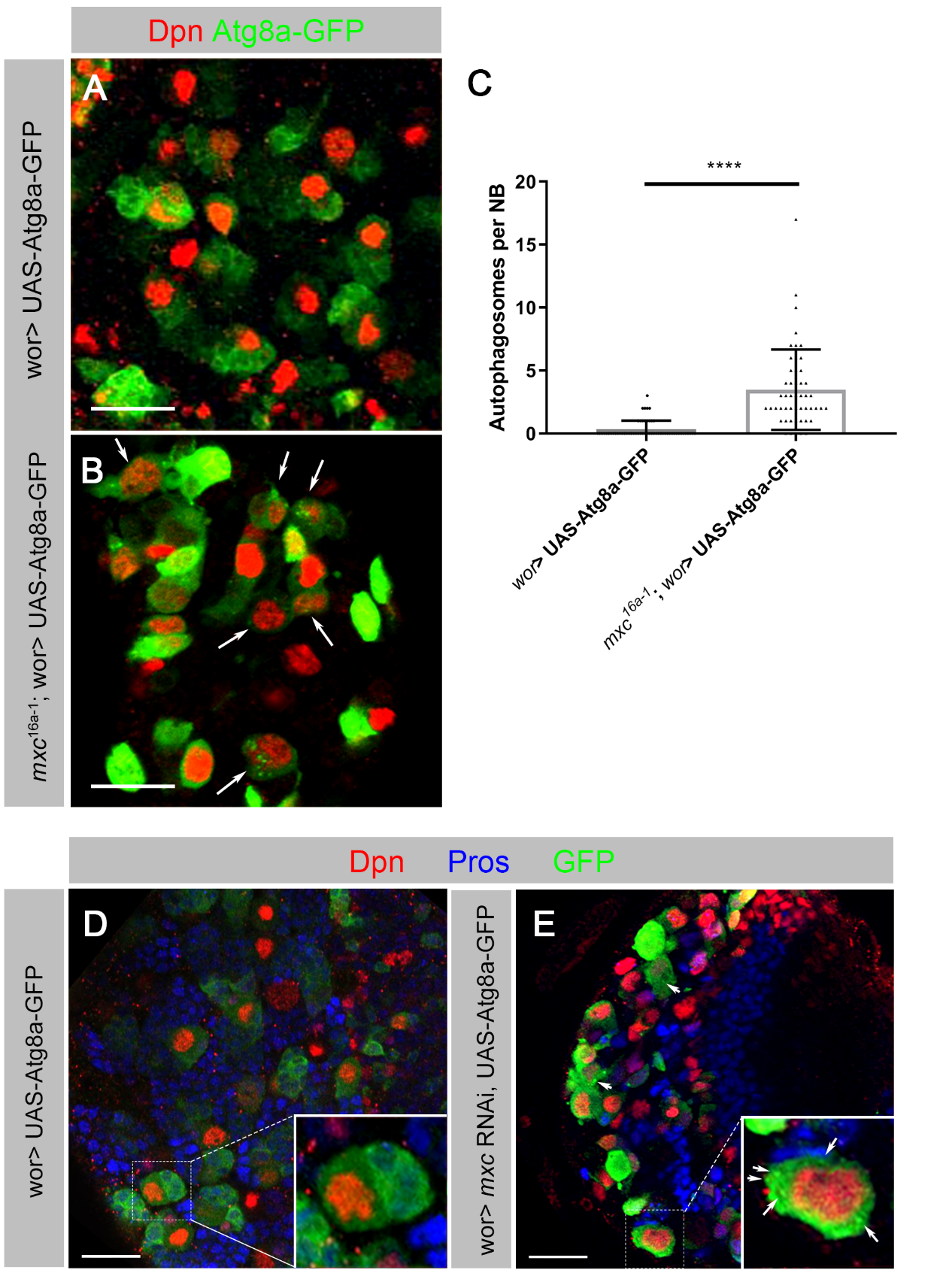


**Figure S6 Autophagy was activated in *mxc* knockdown or mutant NBs**

(A-B) Third‐instar larval brains of *wt* (A) and *mxc^16a-1^* (B), labeled by anti‐Dpn (red) and anti‐GFP (green). These lines contained UAS-Atg8a-GFP driven by *wor*-GAL4. In the *wt* larval central brain (A), only the basic signal of Atg8a-GFP was detected. In the *mxc^16a-1^* larval central brain (B), obvious Atg8a puncta (arrowheads) were detected in the cytoplasm of the NB. Scale bars: 20 μm. (C) Statistical analysis of autophagy levels in the NBs of *wt* and *mxc^16a-1^* larval central brains. The data are plotted as mean ± SD. *****p* < 0.0001 using a Student’s *t* test, *p* = 7.441E-11. Numbers of NBs counted N = 57, N = 50, respectively. (D-E) Third‐instar larval brains of *wt* (D) and *mxc* RNAi (E), labeled by anti‐Dpn (red) and anti‐GFP (green). These lines contained UAS-Atg8a-GFP which was driven by *wor*-GAL4. Obvious Atg8a-GFP puncta (arrowheads) were also detected in the cytoplasm of *mxc* knockdown NB. Scale bars: 20 μm.
